# Structural Aspects of the Allergen-Antibody Interaction

**DOI:** 10.3389/fimmu.2020.02067

**Published:** 2020-09-02

**Authors:** Anna Pomés, Geoffrey A. Mueller, Maksymilian Chruszcz

**Affiliations:** ^1^Indoor Biotechnologies, Inc., Charlottesville, VA, United States; ^2^National Institute of Environmental Health Sciences, Durham, NC, United States; ^3^Department of Chemistry and Biochemistry, University of South Carolina, Columbia, SC, United States

**Keywords:** allergy, allergen, IgE antibody, structure, X-ray crystallography, nuclear magnetic resonance, cryo-electron microscopy, mass spectrometry

## Abstract

The development of allergic disease involves the production of IgE antibodies upon allergen exposure in a process called sensitization. IgE binds to receptors on the surface of mast cells and basophils, and subsequent allergen exposure leads to cross-linking of IgE antibodies and release of cell mediators that cause allergy symptoms. Although this process is quite well-understood, very little is known about the epitopes on the allergen recognized by IgE, despite the importance of the allergen-antibody interaction for the allergic response to occur. This review discusses efforts to analyze allergen-antibody interactions, from the original epitope mapping studies using linear peptides or recombinant allergen fragments, to more sophisticated technologies, such as X-ray crystallography and nuclear magnetic resonance. These state-of-the-art approaches, combined with site-directed mutagenesis, have led to the identification of conformational IgE epitopes. The first structures of an allergen (egg lysozyme) in complex with Fab fragments from IgG antibodies were determined in the 1980s. Since then, IgG has been used as surrogate for IgE, due to the difficulty of obtaining monoclonal IgE antibodies. Technical developments including phage display libraries have contributed to progress in epitope mapping thanks to the isolation of IgE antibody constructs from combinatorial libraries made from peripheral blood mononuclear cells of allergic donors. Most recently, single B cell antibody sequencing and human hybridomas are new breakthrough technologies for finally obtaining human IgE monoclonal antibodies, ideal for epitope mapping. The information on antigenic determinants will facilitate the design of hypoallergens for immunotherapy and the investigation of the fundamental mechanisms of the IgE response.

## Introduction

The interaction between allergens and IgE antibodies is at the core of the allergic response. Epitopes could potentially be located on any part of the allergen surface. However, evidence shows that antibodies are very specific about the epitopes that they recognize and certain areas on the allergen seem to be preferential for antibody binding. The identification of epitopes recognized by IgE is valuable for the design of hypoallergens or other therapeutics. However, allergen-epitope information has been difficult to obtain. This review will discuss various methods to probe epitopes and the knowledge that has been gained from available studies on allergens.

## Historical Perspective to IgE Epitope Mapping

Since the 1980s, efforts to identify antigenic determinants on allergens have been pursued, but progress in the area has been slow due to technical limitations. Original epitope mapping studies were based on the synthesis of overlapping peptides covering the full sequence of the allergen, and the selection of the peptides that bound IgE (1, 2). This approach led to the identification of linear epitopes that comprise a sequential or continuous set of amino acids. However, allergens are proteins or glycoproteins with a defined three-dimensional structure that determines the molecular surface and epitopes recognized by antibodies. Therefore, most allergenic epitopes are conformational, involving amino acids that are close in space due to the protein folding, but non-contiguous in the allergen sequence (3). Technologies that consider the three-dimensional structure of the allergens were necessary to analyze conformational epitopes.

In the absence of complete structural information, most of the original approaches to epitope mapping were indirect, based on the reduction of IgE antibody binding to modified allergen molecules in dot blots or enzyme-linked immunosorbent assays (ELISA) (4). They were possible thanks to peptide synthesis or to the development of recombinant technology, with *in vitro* expression of either allergen fragments, mutants, or allergen chimeras, and their subsequent testing for IgE antibody binding. The development of microarrays or bead-based epitope assays facilitated the investigation of the relevance of linear epitopes, using large sets of linear peptides (5, 6). Microarrays have been especially useful for food allergens because they have mainly linear epitopes due to food processing and/or digestion (6–9). Several IgE/IgG4-binding peptide epitopes were suggested as biomarkers for predicting clinical reactivity and severity to certain foods (10, 11). Another approach uses information from the allergen structure, and hybrid or chimeric allergens are designed by combining the sequences of homologous allergens from different species (12–14). Patches on the allergen surface associated with binding of IgE (from sera of subjects allergic to one of the allergens in the chimera) indicate the presence of epitopes (most likely conformational) on those regions. Another approach to epitope mapping is the identification of mimotopes, which mimic the structure of an epitope (15). It is based on the use of phage display libraries for the selection of peptides that, in combination with a computational algorithm, allow the identification of patches on the allergen surface that mimic conformational epitopes (16, 17). A knowledge of the allergen structure is needed, but the mimotope resulting from the analysis is not necessarily the same as the real epitope. Each of these technologies has provided valuable information on epitope mapping.

X-ray crystallography and nuclear magnetic resonance have determined the three-dimensional structure of many allergens, which helps immensely in interpreting epitopes. Allergens have a wide variety of three-dimensional structures, despite belonging to a limited number of protein families (18–20). Only 1.3% of the total Pfam domains are present in allergens (http://www.meduniwien.ac.at/allfam/). Once the allergen molecular surface is defined, certain amino acids can be selected for site-directed mutagenesis to analyze allergen-antibody interactions (21). Experimental IgE binding and cross-reactivity data can be compared for homologous allergens in conjunction with the molecular structure to understand the approximate location of IgE-binding epitopes (22, 23). Ultimately, the structures of allergen-antibody complexes provide the most detailed information of the epitope-paratope interaction. These precise technologies, although more laborious, directly identify the residues involved in allergen-antibody interactions. This review primarily covers X-ray crystallography and NMR approaches to epitope mapping (Table 1). Additional technologies that also consider the three-dimensional structure of proteins for epitope mapping are cryo-electron microscopy (cryo-EM) and chemical protection assays combined with mass spectrometry (MS). These will also be briefly discussed.

**Table 1:** Comparison of four epitope mapping techniques that consider the three-dimensional structure of the allergen: X-ray crystallography, NMR, cryo-EM and mass spectrometry.

**X-ray crystallography**	**Nuclear magnetic resonance**
• Crystalline state, however, the crystals contain ~30–70% of disordered solvent	• Solution conditions (requires weeks of stability for data collection).
• Theoretically no structure size limit • Proteins purified from natural sources can be used	• High resolution structures up to ~30 kDa.
• Expression with isotope is typically not required for proteins or DNA. Sometimes selenomethionine is incorporated instead of Met.	• Protein/DNA samples usually require ^13^C and ^15^N labeling (stable isotopes). Cost of expression is prohibitive except in prokaryotes.
• X-rays diffraction data are recorded, and the diffraction patterns are used to calculate initial electron density maps. The maps are used to trace a model of the macromolecule, that is later refined and validated	• Data is nuclear resonance frequencies of primarily ^1^H, ^13^C, and ^15^N. Distances between ^1^H atoms are used to build ensembles of possible structures.
• Highly flexible/disordered regions of proteins cannot be modeled and are absent in the final models	• Motion and disorder can be directly measured on many time scales.
**Mass spectrometry**	**Cryo-electron microscopy**
• Typically used in protection assays for epitope mapping. • High sensitivity/low sample requirements. • Atomic resolution identifies specific residues for protection from modification. • Residues that are convenient to modify in protection assays are not always useful for epitope mapping. • Chemistry of modification procedures can have off target effects.	• Can determine atomic resolution structures frozen from solution in vitreous ice. • Low sample requirements. • Resolution occasionally as good as X-ray crystallography. • Performs better on very large samples with high symmetry, typically 100's of kDa, so it is currently not easily or generally applicable to allergen epitope mapping.

## State-of-the-Art Technologies for Epitope Mapping

### X-Ray Crystallography

Over 88% of experimental models of macromolecules that are deposited in the Protein Data Bank (PDB) were determined using X-ray crystallography (Table 1). This technique is often used to generate experimental models of antigen-antibody complexes, and allows for a detailed description of epitopes, paratopes and their chemical interactions. Structural analysis by X-ray crystallography provides the most detailed description of interactions between allergens and antibodies, but is not always easy to perform (24, 25). This approach requires the generation of: (1) significant quantities (mg amounts) of pure and homogeneous protein preparations, specifically the allergen-antibody complex, and (2) a well-diffracting crystal to perform an X-ray diffraction experiment. An additional difficulty in studying epitopes is that highly flexible molecules, like antibodies, are typically recalcitrant to the process of crystallization. To our knowledge, there is not currently a single structure of an antigen in complex with an intact antibody. Fragments derived from monoclonal antibodies (Fab, Fab′) or antibody constructs (single-chain variable fragment -scFv-, scFab, rFab) are used for crystallization because they have significantly reduced conformational flexibility in comparison with intact immunoglobulins. Success in obtaining well-diffracting crystals is not guaranteed, even when sufficient quantities of pure and homogeneous allergen-antibody complexes are available. As crystallization conditions cannot be predicted, hundreds or thousands of trials using different solvent conditions are tested, as well as modifications to the allergen and antibody (26). Once a well-diffracting crystal is obtained, the process of structure determination can be very fast, as currently available software allows to determine initial models very quickly after collection of diffraction data. Therefore, taking into account the many advances in molecular biology, instrumentation, and software development, it is not surprising that the number of experimental structures deposited to the PDB and determined by X-ray crystallography continuously increases. Currently, 145,000 models have been determined using this technique. However, very few are structures of allergen-antibody complexes (see section Structures of Allergen-Antibody Complexes by X-Ray Crystallography).

### Nuclear Magnetic Resonance (NMR)

NMR approaches to observe antibody complexes utilize molecules in solution as opposed to crystallization that attempts to coax molecules out of solution and into a crystal lattice. NMR detects the resonant frequencies of atoms in a magnetic field. These frequencies are primarily influenced by the type of atom (^1^H, ^13^C, or ^15^N) and secondarily by the chemical environment of particular atoms. These data provide a rich source of atomic structure when the resonant frequencies can be specifically attributed to individual atoms (Table 1).

The primary struggle with NMR is sensitivity, which is why large powerful magnets are required. An additional difficulty in observing macromolecules is that the signals become exponentially more difficult to observe as molecular weight increases. NMR methods can determine macromolecular structures but are typically limited to molecules of <20 kDa for high resolution structures. All of the atoms in small allergens (approximately <20 kDa) can be readily observed, while much larger complexes (such as IgE, 190 kDa, combined with two allergens) require specific labeling of certain chemical groups that provide high sensitivity. It is important to realize that, in contrast to crystallography that directly determines the structure of the complex, the NMR data on epitopes requires a careful comparison of the atomic frequencies or intensities in the allergen before and after complexation. Therefore, the NMR results are potentially subject to interpretation in the context of previously known structures or epitope mapping data.

### Cryo-Electron Microscopy (Cryo-EM)

Another emerging methodology that may become applicable to epitope mapping is cryo-electron microscopy (Cryo-EM) (Table 1). Due to technical improvements in the detectors, and secondarily computational methods, cryo-EM has demonstrated the ability to determine macromolecular structures at resolutions occasionally as good as X-ray crystallography, but frequently reasonable for epitope mapping (27). In July 2020 there were 63 Cryo-EM structures with <2 Å reconstruction resolution out of more than 5,000 reported in the PDB. Some attractive advantages of Cryo-EM include that samples are flash frozen, so they don't require crystallization and much less sample is typically required, frequently less than a mg. However, in the sample, the molecules still need to be relatively homogeneous in purity and conformation so the inherent flexibility of antibodies may preclude high resolution analysis. Cryo-EM is the opposite of NMR, regarding its preference of larger molecules for higher resolution information, whereas NMR yields more detailed information on smaller molecules. Although smaller antibody constructs such as Fv or scFv are presently too small for structural analysis by Cryo-EM, this technique may improve to facilitate the use of smaller proteins (28, 29).

There have been several papers on epitopes mapped by Cryo-EM, which are worth noting. For example, Fab fragments from monoclonal antibodies were localized on the spike protein of SARS-CoV-2, and on Zika virus particles (30, 31). More intriguing was the characterization of multiple epitopes simultaneously using polyclonal Fab from sera in a study of neutralizing antibodies of the HIV envelope trimer (32). The study was able to characterize several epitope sites from a small blood volume derived from an immunized animal. Notice that the antigens in all three cases were very large proteins or particles, which is favorable for Cryo-EM characterization but is not typical of allergens which are usually small proteins. However, in this rapidly developing field, studies like these may be feasible in the future for allergens.

### Protection Assays Combined With Mass Spectrometry (MS)

Alternative methods for epitope mapping that rely on mass spectrometry are described in this section (Table 1). They differ from the methods described above that are traditionally associated with structural biology and determination of experimental models of macromolecules. One of the MS approaches is called paratope or epitope “excision” (33). The excision procedure includes enzymatic proteolysis that allows for generation of peptides forming epitopes or paratopes, which later are identified using, for example, a combination of MALDI (matrix-assisted laser desorption/ionization) and ESI (electrospray ionization) mass spectrometry (33–35). This approach requires a small sample that does not need to be labeled. However, as most often the epitopes of interest are discontinuous/conformational in nature, excision is usually combined with chemical modification of the studied complexes. The most common method of the chemical modification involves hydrogen-deuterium exchange (HDX). During this modification the antigen-antibody complex is placed in heavy water and protein backbone amide hydrogen atoms (^1^H) can be exchanged for deuterium (^2^H). The rate of the ^1^H-^2^H exchange (HDX) depends on solvent accessibility and dynamics of a particular protein fragment. Generally, hydrogens that are buried within the protein core or shielded from the solvent, such as hydrogens buried in an antigen-antibody interface will have a low rate of ^1^H-^2^H exchange. After the incubation in heavy water the complex undergoes enzymatic cleavage and the resulting peptides are identified by the change in mass, using MS. It is expected that surface residues forming epitopes and paratopes will have a relatively low level of incorporated ^2^H. This information combined with the molecular models of the antigen and the antibody allows for mapping of the interacting molecular surfaces. Therefore, HDX-MS became a very successful technique that not only has found application in analysis of antigen-antibody complexes, but also in mapping of other protein-ligand interactions (36, 37). Moreover, HDX-MS can be used for studies of protein conformational dynamics, and was successfully used in characterization of the dynamic behavior of antibodies (38, 39).

^1^H-^2^H exchange is not the only chemical modification that can be applied in protection assays combined with mass spectrometry. For example, various surface exposed amino acids can be oxidized by H_2_O_2_ or modified by photochemically induced reactions (40, 41). The modifications to the allergen before and after complexation with the antibody can be compared for epitope information.

## Structures of Allergen-Antibody Complexes by X-Ray Crystallography

The X-ray crystallographic structures of allergen-antibody complexes were first determined for egg lysozyme with fragments of murine IgG monoclonal antibodies (mAb) (42–47). Subsequently, other structures were reported for other allergens, where murine IgG mAb were selected as surrogates for human IgE, due to their capacity to inhibit binding of human IgE antibody to the allergen (Table 2) (48–53, 56, 58, 60–64). These studies involved the purification of an allergen either from the natural source or from *in vitro* cultures expressing recombinant allergens. The IgG mAb were cleaved using pepsin or papain, which resulted in F(ab′)_2_ -that was reduced to F(ab′)- or Fab, respectively. These antibody fragments contain the paratope and were purified and combined with the allergen to form a complex, which was purified for crystallography.

**Table 2:** Structures of allergen-antibody complexes by X-ray crystallography.

**Allergen in complex with IgG antibody construct**					
**Allergen**	**Allergen source**	**Allergen expression system**	**Antibody**	**Antibody expression system**	**PDB code**
Api m 2	Honeybee	Insect cells (high five)	Fab; mIgG1 mAb 21E11	*Mus musculus* hybridoma cells	2J88 (48)^*^
Bet v 1	Birch	*E. coli*	Fab′; mIgG1 mAb BV16	*Mus musculus* hybridoma cells	1FSK (49)^*^
Bla g 2	German cockroach	*P. pastoris*	Fab′, mIgG1 mAb 7C11	*Mus musculus* hybridoma cells	2NR6 (50) (51)^*^
Bla g 2	German cockroach	*P. pastoris*	Fab, mIgG1 mAb 4C3	*Mus musculus* hybridoma cells	3LIZ (52)^*^
Der f 1	House dust mite	*D. farinae* mite culture	Fab; mIgG1 mAb 4C1	*Mus musculus* hybridoma cells	5VPL (53) (54)^*^
Der p 1	House dust mite	*D. pteronyssinus* mite culture	Fab; mIgG1 mAb 4C1	*Mus musculus* hybridoma cells	1) 3RVW (53) 2) 3RVX (53) (55)^*^
Der p 1	House dust mite	*D. pteronyssinus* mite culture	Fab; mIgG1 mAb 5H8	*Mus musculus* hybridoma cells	4PP1 (56) (57)^*^
Der p 1	House dust mite	*D. pteronyssinus* mite culture	Fab; mIgG1 mAb 10B9	*Mus musculus* hybridoma cells	4PP2 (56) (57)^*^ (55)^*^
Der p 2	House dust mite	*P. pastoris*	Fab; mIgG1 mAb 7A1	*Mus musculus* hybridoma cells	6OY4 (58)^*^ (59)^*^
Fel d 1	Cat	CHO	Fab; IgG4 mAb REGN1909	CHO^c^	5VYF (60)
Gal d 4 (lysozyme)	Chicken	Not specified (most likely *Gallus gallus*)		*E. coli* (1FDL) or *Mus musculus* hybridoma cells	(1) (42) (2) (43) (3) 3HFM (44) (4) 1FDL (45) (5) 1MLC (46) (6) 1YQV (47)
Gal d 4 (lysozyme)^#^	Chicken	Not specified	Human V_H_ domain; VH H04 Phage displayed	*E. coli* BL21 Gold	(1) 4PGJ (61) (2) 4U3X (61)
Phl p 7	Timothy grass	*E. coli* BL21 star DE3	Fab; hIgG1 mAb 102.1F10 was expressed based on a hIgG4 that was generated from matched heavy- and light-chain sequences by single B cell cloning from allergic individuals	FreeStyle 293F	5OTJ (62)
**Allergen in complex with IgG antibody constructs containing human IgE variable regions**					
Bos d 5	Cow	*E. coli*	Fab; hIgG1 mAb D1: C_κ_ and CH1 of IgG1 cloned with IgE V_H_/V_L_ isolated from human IgE derived from a combinatorial library	*E. coli* RV308	2R56 (63)
Phl p 2	Timothy grass	*E. coli* BL21	Fab; hIgG1 mAb huMab2: C_κ_ and CH1 of IgG1 cloned with IgE V_H_/V_L_ isolated from human IgE derived from a combinatorial library	CHO-K1	2VXQ (64)^*^

* *Manuscripts that report inhibition of IgE antibody binding by the antibody used in the X-ray crystal structure (or viceversa)*.

# *Only selected complexes with lysozyme are listed. For example, complexes of human V_H_ domains with lysozyme were chosen to compare them with complexes formed by Fabs*.

**Figure 1:**
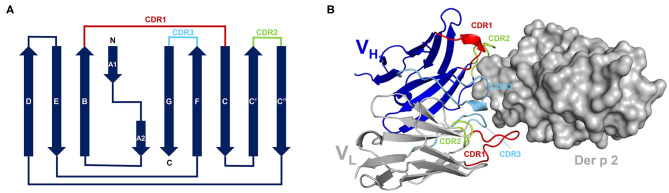
**(A)** A topological diagram of IgG V_H_. β-strands are shown as arrows that indicate direction of the peptide. N- and C-termini, as well as individual β-strands are labeled. Loops corresponding to three CDRs are highlighted using different colors. The figure was prepared based on a diagram presented by Bodelón et al. (65). **(B)** Complex between 7A1 and Der p 2.0103. Only variable domains of the 7A1 antibody are shown. CDRs are marked using the same colors as used for the topological diagram. Der p 2.0103 is shown in surface representation.

Although there are <20 different allergens that have their structures determined in complexes with antibodies (Table 2) their analysis provides interesting insights into epitopes and paratopes. Chicken lysozyme (Gal d 4) is often used as a test molecule and there is a vast amount of literature on the use of this protein to study interactions with antibodies. Therefore, to avoid bias that a large number of lysozyme-antibody complex may cause we selected for analyses only some representative structures. While allergens have a wide variety of structures, antibodies of the same isotype have the same structure, formed by immunoglobulin-fold domains of about 100 amino acids. Light chains have an N-terminal variable domain (V_L_) followed by a constant domain. Similarly, heavy chains have a variable N-terminal domain (V_H_), but it is followed by either 3 (in IgG) or 4 (in IgE) constant domains. The central part of these domains is made of anti-parallel β-sheets, in which β-strands are linked to form the so-called Greek-key motifs. For example, IgG V_H_ domain is made of anti-parallel β-sheets composed of nine β-strands that are linked by eight loops (Figure 1) (65). Of the four apical loops, only loops 1, 2, and 4 interact with the antigen, and contain the complementary determining regions (CDRs): CDR1, CDR2, and CDR3, respectively. The loops 1, 2, and 4 are between beta-sheets B-C, C′-C″, and F-G, respectively (Figure 1). A similar β-sheet configuration occurs in the light chain. The 6 loops involved in paratope formation form the following CDRs: H CDR1, H CDR2, H CDR3 in the heavy chain, and L CDR1, L CDR2, and L CDR3 in the light chain. The CDRs contain the amino acids that form the paratope. The H CDR3 is sufficient for most antibody specificities (66), although exceptions have been found (67). In a few cases, additional residues outside the CDR, located in the “framework” of the antibody, can also contribute to antibody binding. While epitopes can be located on different parts of the allergen surface, paratopes are always at the apical region of the variable domain of the antibody, formed by the 6 CDRs. The CDR boundaries have been historically defined in different ways (68–70), and currently the ImmunoGenetics website (www.imgt.org) utilizes a consensus for their estimation.

**Figure 2:**
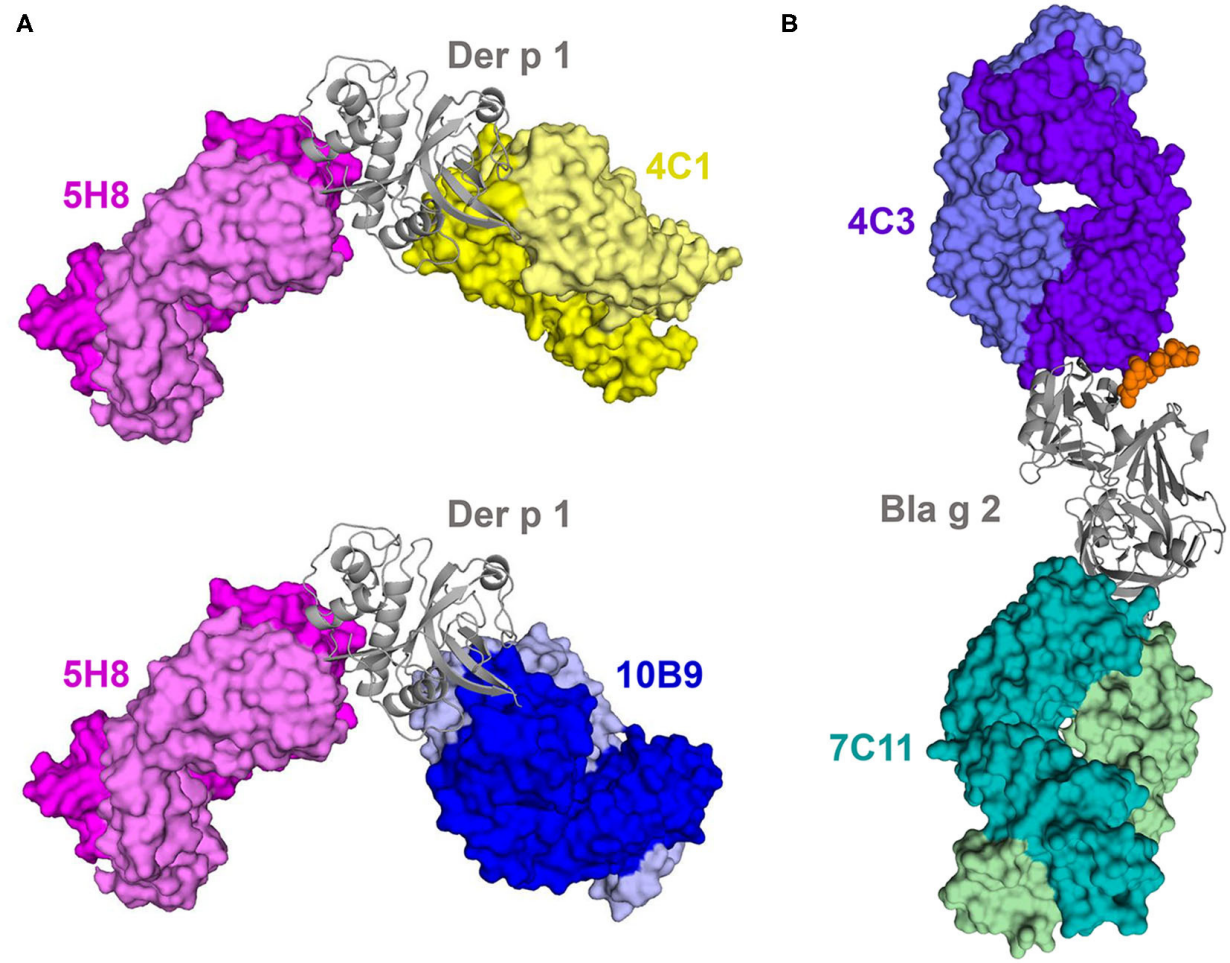
Cartoon representations of complexes between antibody Fab fragments and allergens Der p 1 **(A)** and Bla g 2 **(B)**. Structures of complexes with antibodies were superposed to compare location of epitopes. Epitopes on Der p 1 for mAb 4C1 and 10B9 partially overlap, but they both are far from the epitope recognized by 5H8. Epitope on Bla g 2 that is recognized by antibody 4C3 included a carbohydrate (shown here as orange spheres). Fab fragments of the antibodies are shown in space-filling models, and allergens are shown using ribbon representations. Light chains are marked using lighter colors.

A significant fraction of the available allergen-antibody structures corresponds to complexes of Group 1—the best studied—and Group 2 house dust mite allergens, and the cockroach allergen Bla g 2 (Figure 2). Groups 1 and 2 comprise the most important major allergens from house dust mites. A major allergen is one to which >50% of subjects allergic to the allergen source are sensitized. Group 1 includes Der p 1, Der f 1, Blo t 1, and others, and are cysteine proteases. Group 2 includes Der p 2, Der f 2, Blo t 2, and others with an MD-2-related lipid-recognition (ML) domain (www.allergen.org). Three X-ray crystal structures of Der p 1 have been determined in complexes with three different murine IgG mAb (4C1, 5H8, and 10B9), from which mAb 4C1 is a cross-reacting antibody that also binds to Der f 1 (53, 56). Comparison of Der f 1 and Der p 1 structures with 4C1 revealed that the cross-reactive mAb binds to a conserved surface patch that is present on both allergens (53). Unexpectedly, this patch is not a part of the largest conserved surface area in common for both Der f 1 and Der p 1, and which includes the active site of the enzymes. The majority of the amino acids forming the central part of the epitope are conserved, and in very similar conformations. Interestingly, the epitopes for 10B9 and 4C1 partially overlap, but 10B9 is not able to bind to Der f 1. The epitope for 4C1 is “rotated counterclockwise” by ~90° in relation to the position of the 10B9 epitope on Der p 1. On the other hand, the 5H8 binding epitope is located at a significant distance from both 4C1 and 10B9 epitopes. The image of the structure of three Der p 1-antibody complexes clearly illustrates that mAb 5H8 and 4C1 or 10B9 can simultaneously bind to the same allergen molecule (Figure 2) (56). Bla g 2 has a bilobal structure typical of aspartic proteases, but it is enzymatically inactive due to substitutions in the catalytic site (71, 72). Two structures of Bla g 2 with mAb 7C11 and 4C3 have been determined, showing their binding to opposite lobes of the molecule (50, 52). The complex with mAb 4C3 was unique because it showed that carbohydrates contributed to the interactions with Bla g 2 (Figure 2). The murine IgG mAbs used in these crystallographic studies were chosen as surrogate IgE antibodies, because they inhibit binding of IgE to the allergen. Once the IgG epitope was identified, site-directed mutagenesis of the allergen residues involved in antibody recognition was performed, followed by IgE antibody binding analysis of the mutants, to identify IgE antibody binding sites. This approach resulted in the design and production of allergen mutants with decreased capacity to bind IgE, which are being investigated as future candidates for immunotherapy (51, 58, 67, 73).

The antibodies in the majority of allergen-antibody complexes that have their structures determined are IgG1. However, three of the structures reported in Table 2 are different from the IgG1 isotype: 1) IgG4 (in complex with Fel d 1), 2) an IgG1 construct engineered to combine the constant domains of human IgG1 heavy and kappa light chains with variable regions of a human IgE construct derived from an scFv combinatorial library (in complex with Bos d 5), or 3) an Fab isolated from a combinatorial library, which is a hybrid of the variable domain of the IgE Fab and the constant domain of human IgG1 (in complex with Phl p 2) (60, 63, 64). The IgG4 (REGN1909) binding Fel d 1 is a fully humanized antibody that was derived from mice immunized with recombinant dimeric Fel d 1 (60). REGN1909 is able to partially block IgE binding to natural Fel d 1, with a maximum inhibition of 51%. REGN1909 together with another IgG4 (REGN1908), which binds to a different non-overlapping epitope, was able to block up to 83% of IgE binding to natural Fel d 1. A combination of X-ray crystallography and HDX-MS was used to elucidate information on the antibody binding epitopes for REGN1908 and REGN1909. Only the crystal structure of Fel d 1 in complex with REGN1908 was obtained (60).

While most often the antibodies that are used in studies of allergens are composed of light and heavy chains with six total CDRs, there are also examples of heavy chain only antibodies. These can contain two heavy chains only (and therefore have 3CDRs for recognition per chain) or single domain antibodies, which have a single antigen binding domain (74–76). Heavy chain only antibodies are present in nature and are produced by camelids and sharks. The paratopes formed by the single chain antibodies have a very similar amino acid composition to that observed in conventional antibodies (77, 78). The heavy chain only antibodies, and especially their V_H_ domains, are relatively easy to produce and their biophysical, as well as structural properties, allow for easy application in biotechnology and therapeutics (77, 79). Single domain antibodies (specific for lysozyme) were isolated years before discovery of heavy chain antibodies in camelids (76), and were proposed as alternative to conventional monoclonal antibodies. Later on, camelids' V_H_ domains also became a model for the generation of their human equivalents. Fully human V_H_ single domains were used to generate complexes with Gal d 4 (Table 2) (61).

**Figure 3:**
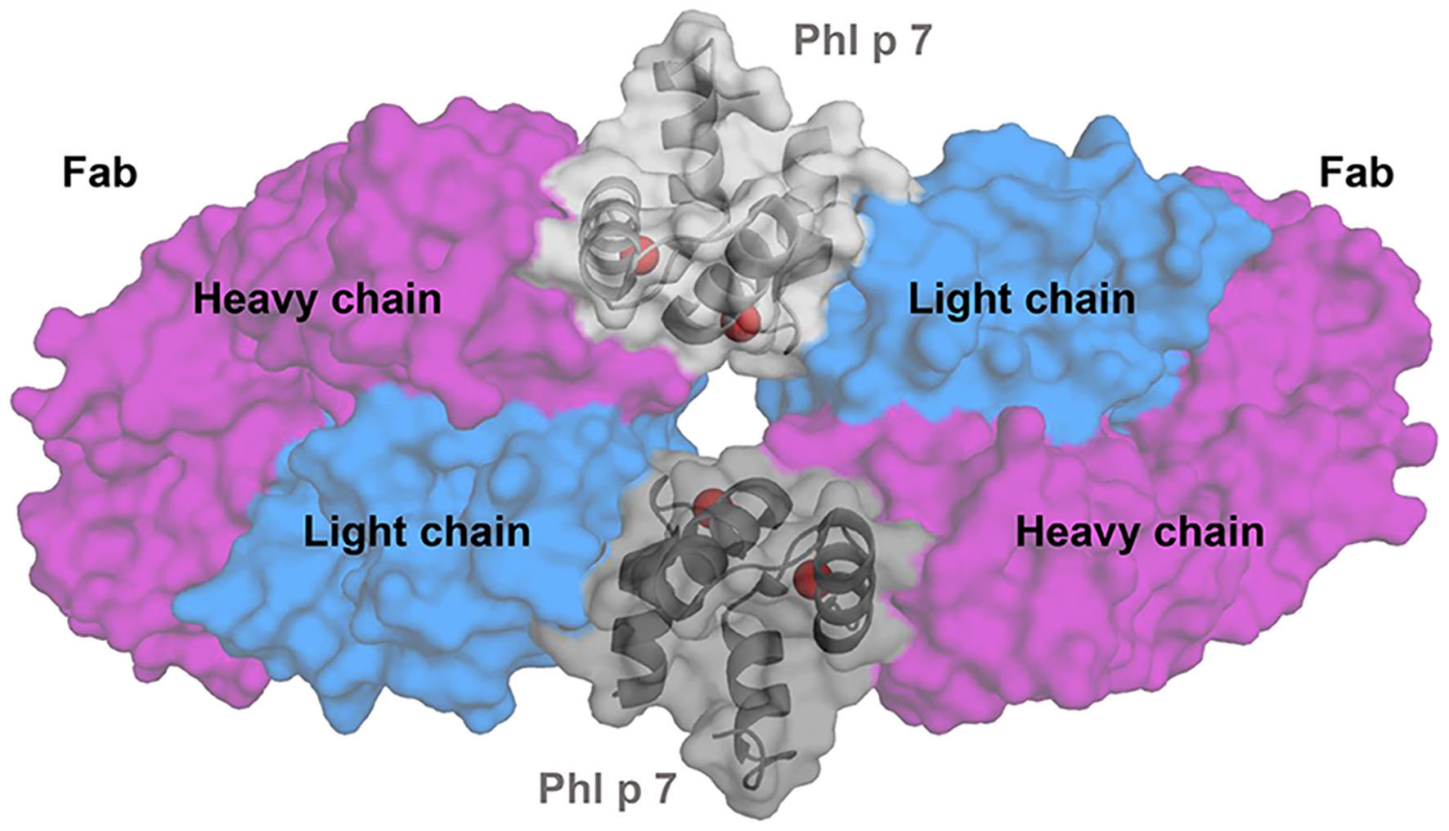
Superantigen Phl p 7 interactions with Fab. Cartoon representation of a complex between two Fab fragments of a human antibody and two molecules of timothy grass pollen allergen Phl p 7 (PDB code: 5OTJ). The crystal structure revealed an unusual binding of two molecules of the monomeric allergen and two molecules of the antibody. Phl p 7 molecules are shown in gray. Light chains of the antibody are shown in blue and heavy chains in purple. Calcium ions bound by the allergen are presented as red spheres.

A new type of allergen-antibody interaction was recently reported thanks to the determination of a Phl p 7-antibody crystal structure (Figure 3) (62). An IgG4 originally generated from single B cell cloning was converted into an IgG1 for structure determination of the allergen-antibody complex. The structure revealed that two antibodies bind simultaneously to Phl p 7 in two different ways: (1) the classical mode that involves both heavy and light chains of the antibody, and (2) an unusual non-standard way, involving only binding of the light chain to the allergen to a separate Phl p 7. This resulted in trapping two monomeric allergen molecules between two molecules of the same antibody (62). While Phl p 7 was not a dimer, the stoichiometry of the complex still required two Phl p 7 molecules. Therefore, this Phl p 7-antibody structure has changed the prior view that one antibody is able to recognize only a single epitope on an allergen/antigen.

The allergens that we have described are proteins, and their interactions with antibodies are the same as for other proteinaceous antigens. It also has to be stressed that X-ray crystallography provides generally a static picture of the interacting molecules. However, both antigens and specifically antibodies display a great level of conformational flexibility (80, 81). It was shown that conformational flexibility and local structural dynamics of antibodies play a very important role in recognition and binding (82, 83). A higher level of conformational flexibility usually is attributed to antibodies that are not matured, and the flexibility allows them to recognize more antigens and/or altered antigens (84). During an antibody's maturation the increase of specificity is often achieved at the cost of the conformational flexibility, and a more rigid antibody binds better to one antigen (85, 86). Therefore, studies of the CDR conformations are critical for understanding the process of recognition and binding of antigens by antibodies (87–90), and these studies are most often performed using NMR, HDX-MS and various computational methods.

## Lessons Learned About Allergen-Antibody Interactions Using X-Ray Crystallography

**Figure 4:**
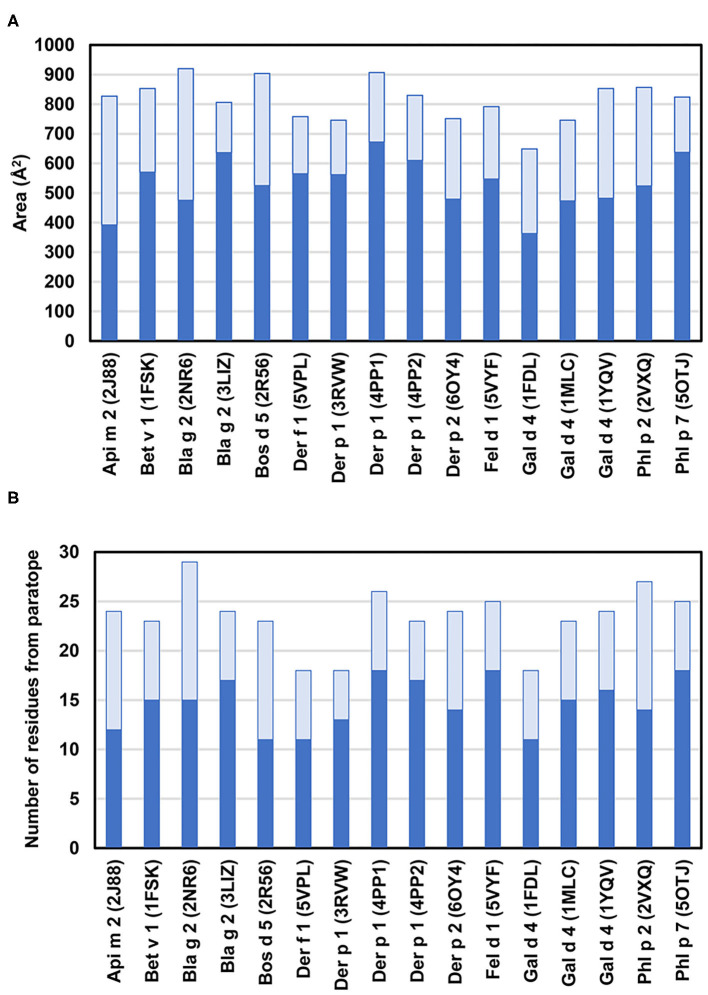
**(A)** Allergen-antibody interface areas. Dark blue color corresponds to the area of the interface that corresponds to heavy chain and light blue color indicates the area of interaction with light chain. In the case of Phl p 7 (PDB code: 5OTJ) the only area corresponding to the standard mode of binding is reported. **(B)** Number of residues from heavy chain (blue) and light chain (light blue) that participate in interactions with allergens. Only residues that contribute at least 2.0 Å^2^ to the interface area (as calculated with PDBePISA) (92) are counted.

An analysis of 16 allergen-antibody structures selected from Table 2 revealed a detailed description of the interface formed by epitopes and paratopes (45–50, 52, 53, 56, 58, 60–64). Typically, the interface area falls in the 650–920 Å^2^ range (an average of 813 Å^2^; Figure 4A) (91). In the complex with Phl p 7 mentioned above, the interface area is larger, and can be divided between a “classic” interface (~820 Å^2^) with one antibody, and an additional interface (~380 Å^2^) responsible for the non-standard interaction with the light chain from the second antibody (62). In most cases, the antibody heavy chain provides the largest contribution toward the total area of the interface, but this contribution is not always significantly bigger than the light chain share (Figure 4A). The light chain provides between 23 and 53% of the interface area. In the Api m 2 and Bla g 2 (2NR6) complexes, the light and heavy chains contribute almost equally to the interface area (48, 50). However, it is worthwhile to note that in two complexes of antibody V_H_ domains with Gal d 4 (PDB codes: 4PGJ and 4U3X), the allergen-antibody interface areas are quite large (810 and 826 Å^2^, respectively), perhaps to compensate that the paratope is formed only by the heavy chain.

**Figure 5:**
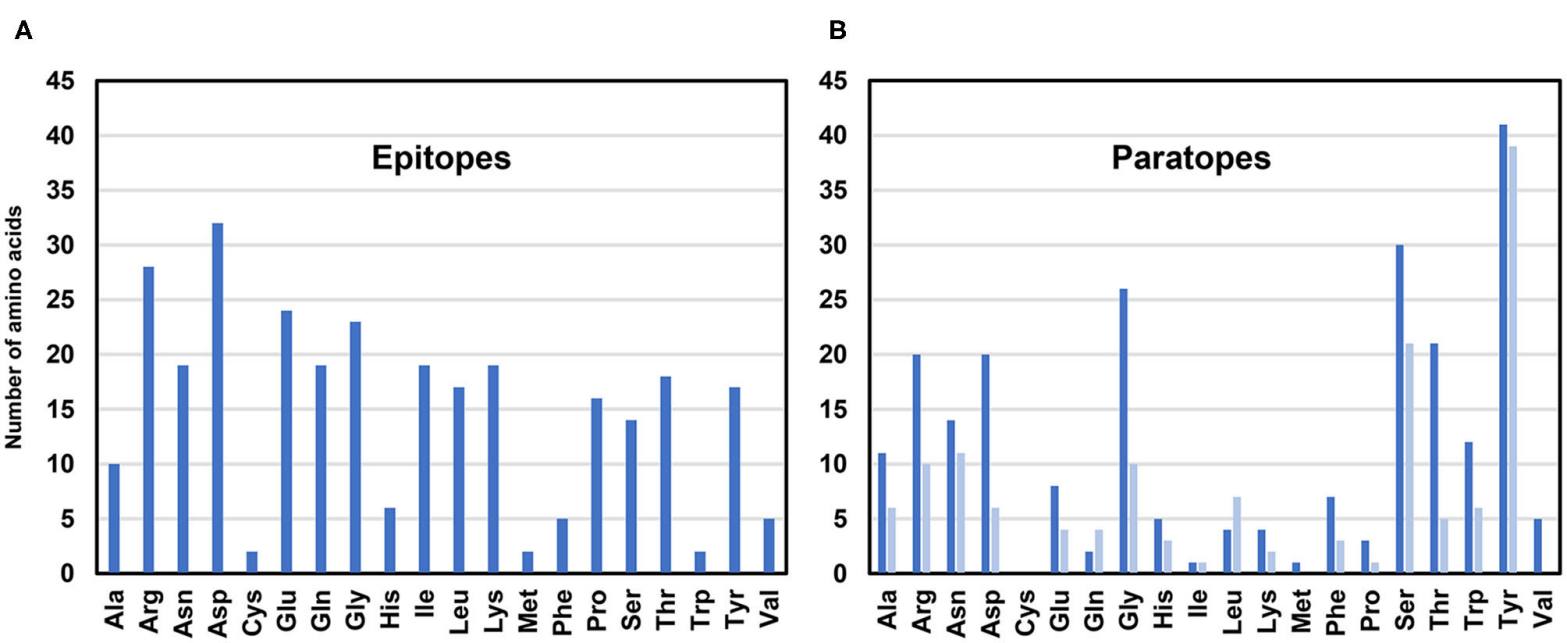
Number of amino acids in epitopes **(A)** and paratopes **(B)**. Data for paratopes is shown for heavy chains (blue) and light chains (light blue). In the case of the Phl p 7 (PDB code: 5OTJ) only residues participating in the standard mode of binding are counted.

**Figure 6:**
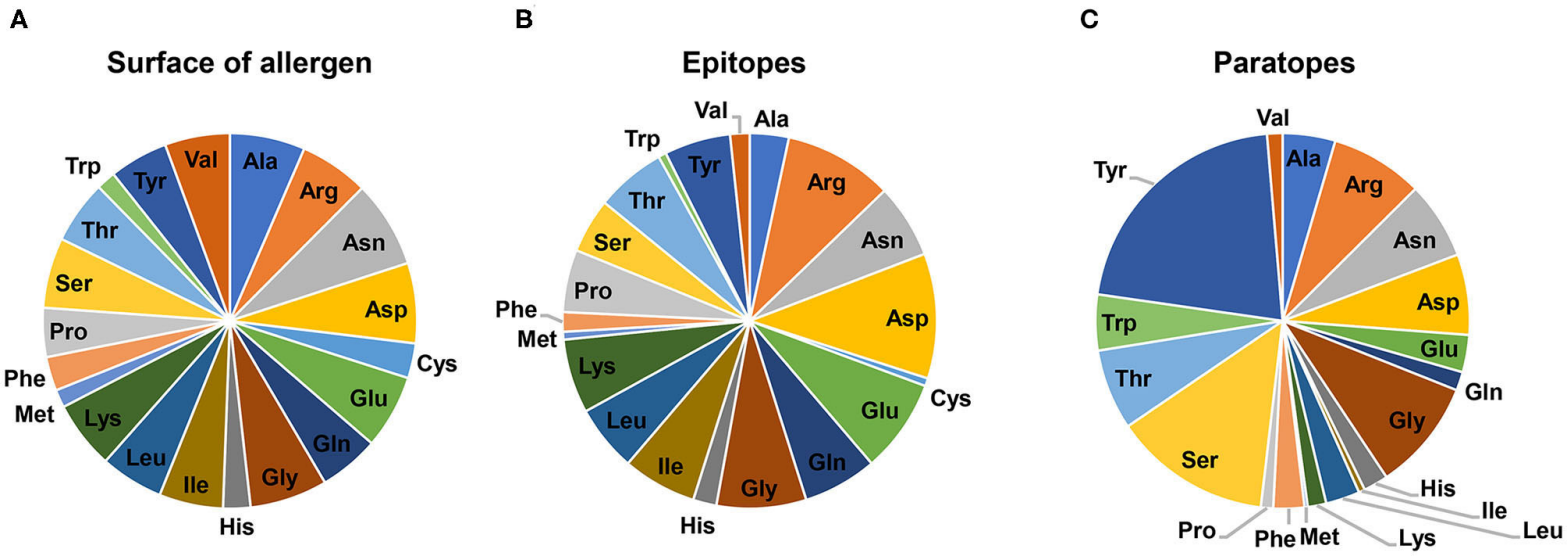
Distribution of amino acids on the surface of allergens listed in Table 2 **(A)**, in epitopes **(B)** and paratopes **(C)**.

**Figure 7:**
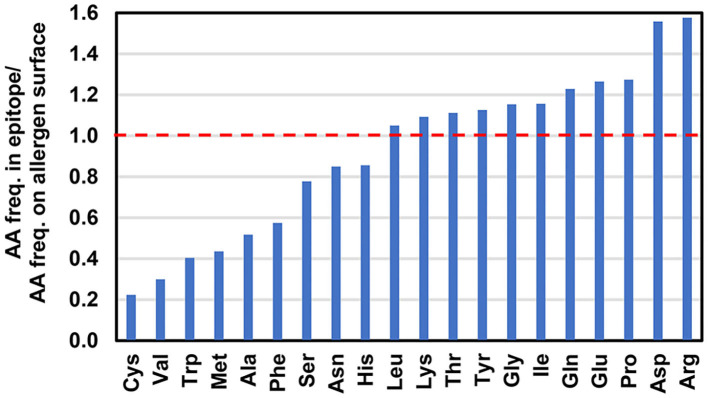
Ratio of amino acid frequencies (AA freq.) in the epitopes to the amino acid frequencies on allergen surfaces. A ratio value above 1 indicates that a particular amino acid is observed more often in the epitopes in comparison with the allergen surfaces.

Analysis of the allergen-antibody interfaces at the amino acid level shows that paratopes are formed by 18–28 residues that interact with epitopes composed of a similar number of amino acids (12–25 in the set of 16 complexes analyzed here) (Figure 4B, Table 2) (45–50, 52, 53, 56, 58, 60–64). In addition, it is possible to examine the distribution of various amino acids in the epitope and paratope areas. While the amino acid composition of epitopes is barely different from the overall composition of the allergen surface residues (93), there is a significant bias in amino acid composition of paratopes (Figures 5–7). Namely, the paratopes have a very high content of tyrosine, serine, and glycine residues, with relatively low content of isoleucine, leucine, lysine, methionine, and proline (94–97). The paratopes also tend to have a relatively high content of aromatic residues (Tyr, Trp, Phe, and His). Unfortunately, the relatively small number of determined structures of allergen-antibody complexes does not allow for generalizations on the compositional bias of allergen epitopes, especially when among the 16 structures analyzed here, three contain Der p 1, three contain Gal d 4, and two contain Bla g 2. However, in large datasets of protein-protein interactions, aromatic residues are also generally favored (98, 99). No obvious differences were observed between allergen-antibody interactions and the antibody recognition of other non-allergen proteins.

**Figure 8:**
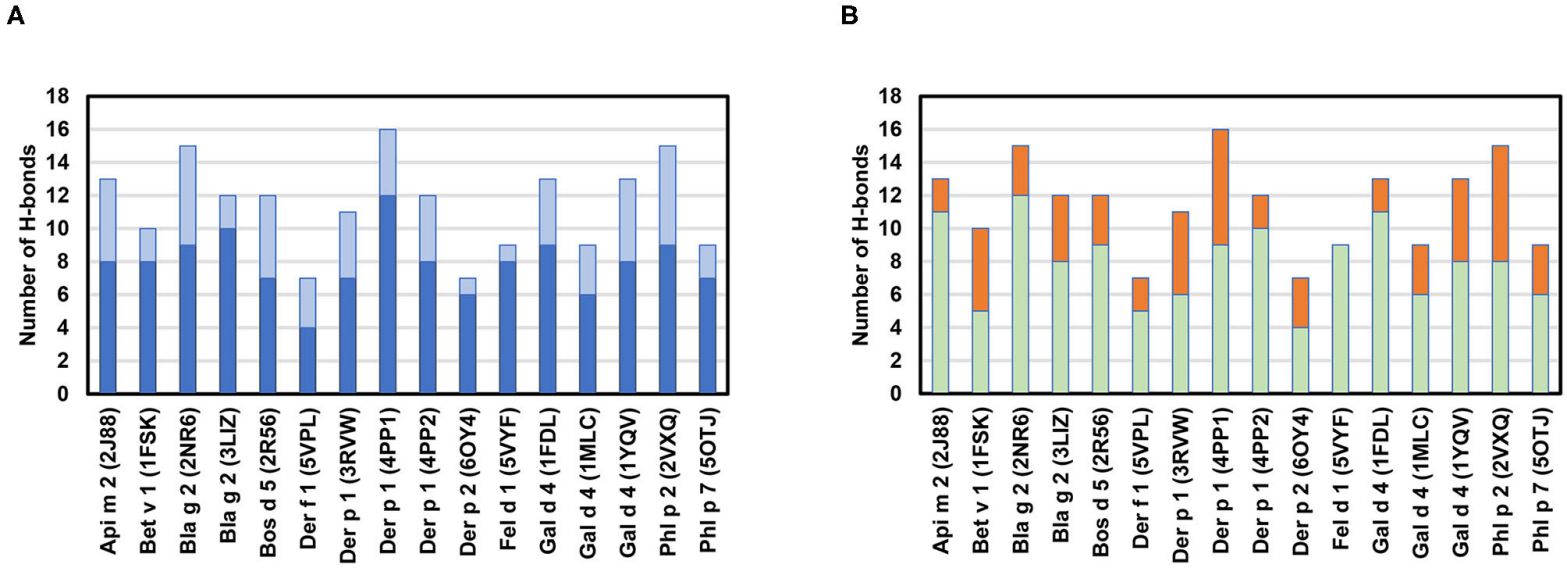
**(A)** Number of H-bonds between paratopes and epitopes. H-bonds formed by residues from heavy chains are indicated in blue and H-bonds formed by residues from light chains are in light blue. **(B)** Number of hydrogen bonds formed by side chain (green) or main chain atoms (orange) of the antibodies. Calculations were made with PDBePISA (92), and only H-bonds for which distances between donor and acceptors were below 3.3 Å are taken into consideration.

The chemical interactions that drive allergen-antibody formation include covalent (H-bonds) as well as non-covalent binding interactions (e.g., hydrophobic, van der Waals, charge-charge, and cation-π interactions). Hydrophobic and electrostatic interactions are most important for a primary contact between antigens and antibodies (100). However, once the distance between antigen and antibody is shortened, van der Waals interactions and H-bonds start to play a significant role. H-bonds are especially important, as they quite often are associated with specificity of the binding. The analysis of 16 structures in Table 2 indicates that there are between 7 and 16 H-bonds that mediate contacts within the epitope-paratope interface (Figure 8). Heavy chains of the antibodies are responsible for the majority of the hydrogen bonds that are formed. While most often atoms that are hydrogen donors or acceptors in the H-bonds belong to the side chains of amino acids forming paratopes or epitopes, there are also hydrogen bonds formed by main chain atoms (Figure 8).

Amino acids with charged side chains also play an important role in mediation of epitope-paratope interactions (101). For example, salt bridges in the interfaces are formed between positively charged amino acids (Arg or Lys) and negatively charged side chains of Asp or Glu. Enrichment of epitopes in such amino acids is illustrated in Figures 5–7. It has been shown that electrostatic interactions increase the binding specificity between antigens and antibodies (102, 103). In addition, positively charged side chains may participate in cation-π interactions (50, 104, 105). This type of interaction is relatively common in antigen-antibody interfaces, as it is formed by aromatic residues (e.g., Phe, Tyr, Trp) and side chains of Arg or Lys, which are over-represented in epitopes and paratopes. For example, cation-π interactions were observed in interfaces formed between Bla g 2 and mAb 7C11, Der p 1 and 5H8, as well as between Der p 2 and mAb 7A1 (50, 56, 58). Side chains of aromatic residues may be also involved in various π-π interactions (56, 105).

Besides protein-protein contacts at the allergen-antibody interface, other chemical moieties can form contacts between the two molecules. One of them is carbohydrates. An epitope on Bla g 2 that is recognized by mAb 4C3 includes a glycan (Figure 2) (52). The role of this glycan as part of the epitope has also been demonstrated in relation to IgE binding and basophil histamine release (106). This observation stresses the potential importance of post-translational modifications of the allergens for their interactions with antibodies (107, 108). Production of recombinant proteins in some systems, such as *E. coli*, which do not add carbohydrates to expressed proteins, may lead to a lack of proper recognition by antibodies from allergic individuals raised against the glycosylated natural allergen. Nevertheless, the allergen recognition might still occur, if the antibody recognizes the protein part of the epitope.

During the process of antigen-antibody recognition many water molecules that were on the surface of the interreacting molecules are displaced. However, this process does not always lead to their complete displacement. In fact, water molecules can play an important role in allergen-antibody interactions, in purely protein-protein complexes, and the aforementioned protein-carbohydrate complex (52). Very often, the water molecules are buried between the allergen and the antibody and mediate the contact between the macromolecules through hydrogen bonds (52, 58, 109). In some cases, the presence of buried water molecules significantly improves the fit between allergen and antibody surfaces, allowing for stronger binding (75).

## Identification of IgE Antibody Binding Epitopes

The most interesting complexes for allergy research are with human IgE, but they are also the most challenging to obtain. One of the main limitations to defining epitopes for human IgE has been the difficulty of obtaining human IgE monoclonal antibodies in the amounts required for crystallography or NMR. IgE is polyclonal and present in low concentrations in blood (ng/mL). B cells expressing IgE circulate in low frequency in peripheral blood (3 × 10^−7^ to 7 × 10^−6^) (110), which makes it difficult to isolate and grow them in primary cultures. Historically, several alternative methods were developed.

One approach to study the human IgE repertoire is to isolate IgE antibody constructs from phage display combinatorial libraries prepared using peripheral blood mononuclear cells (PBMC) of allergic subjects (111–113). Basically, antibody heavy chains are combined with light chains from the same or a different subject, to form IgE antibody constructs that are displayed by phagemids. These constructs are then isolated based on their allergen specificity in a selection process called panning. Such technology relies on the fact that antibody specificity largely resides in the heavy chain variable domain and its third hypervariable loop (H CDR3) (66). Phage display technology led to the isolation of IgG1 antibody constructs with IgE variable domains against Bos d 5 from cow and Phl p 2 from timothy grass pollen, and the allergen-antibody Fab complexes were determined by X-ray crystallography (Table 2) (63, 64). Since the antibodies were isolated using IgE combinatorial libraries, it is not known whether light and heavy chain pairing corresponds to that observed in antibodies produced by allergic individuals. Both structures are useful, as they currently provide the closest picture of the interactions between allergens and IgE that take place in humans.

The structure of the Bos d 5-antibody complex illustrates an additional important phenomenon, namely the importance of the oligomerization state or quaternary structure of the allergen. Bos d 5 is a dimer in the reported structure (114). Dimerization of an allergen allows for cross-linking of IgE receptors with the same antibody. Localization of IgE epitopes clearly illustrates why, for allergens forming homo-oligomers, only one epitope per protein chain is sufficient for the allergic reaction to be triggered (115, 116). For example, a cockroach Bla g 2 mutant with amino acid substitutions that prevented dimerization induced less β-hexosaminidase release from mast cells than the dimeric wild-type Bla g 2, suggesting a functional role of dimerization in allergenicity (50). Dimerization of an allergen also provides an opportunity to use a single mAb binding for capture and detection in a “sandwich” ELISA (115, 117).

Other studies addressed allergen epitope mapping using indirect approaches. A cluster of several IgE antibody binding epitopes was located on the C-terminal domain of Phl p 1 using human IgE obtained by phage display technology. In combination with site-directed mutagenesis, the authors designed a hypoallergenic group 1 grass pollen allergen fragment (118). Two other studies used IgE constructs from phage display libraries to map epitopes on Phl p 5 and Bet v 1 (119, 120). Four independent epitope clusters on Phl p 5.0101 and two on Phl p 5.0201 were identified (119). Four Bet v 1-specific IgE (for one of which the structure was determined) were identified that targeted two non-overlapping epitopes in Bet v 1, as assessed by immunological assays (120).

Recently, a house dust mite Der p 2-specific IgG mAb overlapping with IgE was mapped by X-ray crystallography and site-directed mutagenesis analysis. A Der p 2-specific IgE construct isolated from a single-chain variable fragment (scFv)-encoding phagemid library recognized the same main residues as the IgG, further confirming the relevance of this epitope to human health (58). These studies underline the utility of using constructs derived from phage display technology to investigate the antigenic determinants relevant to allergy.

Alternative approaches to isolate antibodies are based on sorting single B cells for amplification of mRNA that encodes for the antibody. They have proven effective for identifying the exact pairing of IgG heavy and light chains, but not for B cells expressing IgE due to their low frequency in blood (121). A study used single B cell RT-PCR to obtain allergen-specific IgG antibody pairings (122). In addition, heavy chain variable gene sequences of IgE antibodies were obtained by deep sequencing PBMCs, but this study did not lead to the production of allergen-specific native pairs for IgE. One recent publication reports single B cell sorting combined with RNAseq as an approach to obtain human IgE mAb against peanut allergens (123). However, large amounts of sequencing (currently at very high cost) would be required to obtain sequences of the full IgE repertoire using this technology.

A new approach to isolate human IgE monoclonal antibodies has emerged using hybridoma technology (124). Individuals are selected according to their specific IgE sensitization, and their B cells are screened for allergen-specific IgE reactivity before fusion with myeloma cells to create hybridomas. This is an advantage versus the RT-PCR approach, in which the allergen-specificity is not known until recombinant antibodies are expressed based on the sequences obtained. Using this technology, several allergen-specific antibodies were isolated and are being used for IgE epitope mapping by X-ray crystallography and NMR (125–127). It should be noted that this method is still labor intensive, but compared to the other approaches, the clones contain the natural pairing of the heavy and light chains increasing the relevance of this technology.

## Epitopes Defined by Nuclear Magnetic Resonance (NMR)

Because of the limitations in the size of proteins for which NMR can determine structures, NMR experiments to determine allergen epitopes necessarily involve clever experimental design and accurate interpretation. In well-designed experiments, the data provides atom-specific information on the epitope region, which can be readily understood in the context of the allergen structure. The following section describes the design and range of applicability of various NMR designed experiments.

### NMR Protection Assays

The earliest NMR epitope mapping experiments designed by Yvonne Paterson and co-workers were protection assays that measured the exchange rate of amide protons for deuterons in an antigen with and without the antibody present, similar to the HDX-MS (128). Instead of measuring a change in mass, the approach takes advantage of the fact that protons and deuterons resonate at very different frequencies. The exchange of protons for deuterons leads to a disappearance of observable ^1^H frequencies in the antigen. In the Paterson design, the antibody was covalently linked to beads to make an affinity column. Subsequently, the antigen in solution was added to the column and allowed to bind the antibody. The buffer was then easily changed from ^1^H_2_O to ^2^H_2_O, so the ^1^H-amide protons on the antigen surface could be exchanged for deuterons, except in the epitope that was protected by the antibody. Finally, the antigen was eluted at low pH to quench or stop further amide exchange. The cleverness of this design is that the antigen (smaller than the antibody, and therefore with better NMR properties) retains information about the protection. Paterson applied this method to the model antigen cytochrome c, and it was subsequently adapted for the allergens hen-egg lysozyme (Gal d 4) and Der p 2 (59, 129).

Each of these protection studies provided useful epitope mapping information for the antibodies analyzed. Paterson showed that one antibody protected from amide exchange 11 residues that were derived from 3 discontinuous peptides (128). The 3 peptides were all in close proximity on the crystal structure. The Der p 2 studies probed the epitopes of 3 murine mAb, one of which (epitope for mAb 7A1) was recently corroborated with a crystal structure, and further NMR data (see below) (58). However, only the protection assays for mAb 7A1 gave discontinuous epitope information. The absence of protection information for the other two antibodies does not imply that the other epitopes are linear. Instead, it was probably due to unfortunate circumstances where the exchange rate for protected versus non-protected was too fast to measure using the antibody column technology.

Additionally, in the lysozyme studies, it became apparent that not only could amides in the epitope be protected from exchange, but more distal atoms could show differences in exchange rate (129). This is understood to be due to conformational changes in the antigen, or changes in the folding-unfolding rate of the protein due to the formation of the complex with the antibody, which was also noticed by Paterson et al. (130). Interestingly, similar distal changes in exchange rate were observed for binding of the Fv fragment of the lysozyme antibody and the mAb 7A1 (58, 131). This is an important lesson for all NMR studies: proximal and distal changes in antigen conformation upon antibody binding can similarly influence the data, and it might be difficult to differentiate *a priori* which changes occur within the epitope. Therefore, it is frequently important to support the observed changes in the NMR data with additional information. This additional data could be proximity in the structure of atoms that experience NMR spectral changes, or data from mutant proteins and complimentary immunoassays to prove or disprove antibody binding.

The HDX-NMR protection assays were successful but have fallen out of favor for several reasons. First, creating an antibody column with enough capacity for an NMR experiment, typically 5–10 mg of antibody, can be cost prohibitive. Second, not all antigens survive the low pH required to quench the exchange. Third, NMR instrumentation improved and labeling techniques (132) with newer experiments [called TROSY (133, 134)] were developed to better observe larger complexes directly, obviating the need of using the antibody column and the measurement of exchange rates.

### NMR Direct Observation of Complexes

The “antibody”-allergen complex can now be directly observed with careful choices of the labeling scheme. Complete ^15^N backbone labeling of the allergen or antibody fragment such as Fab (~50 kDa) or scFv (~25 kDa) is sometimes possible. These amide detection techniques using antibody fragments have been successfully applied to the allergen Blo t 5 in complex with a Fab. A discontinuous epitope was identified by comparing the ^1^H-^15^N chemical shift perturbations of the bound and free allergen (135, 136). This epitope was shown to overlap with binding sites of patient polyclonal IgE. However, in our experience, the use of smaller forms of the whole antibody, such as Fab or scFv, in an attempt to increase NMR signals, has not always been successful. Some of these smaller antibody constructs are hard to produce, and surprisingly, do not always maintain the high affinity of the full antibody for the antigen. Therefore, other techniques such as those below have been explored for NMR detection of allergen-antibody complexes.

A similar labeling scheme, but in a subtly different experiment, was used to map the Der f 2 epitopes of two full length murine IgG (150 kDa) (137). The authors again utilized ^1^H-^15^N labeled allergen, which would typically not be detectable at this large size when bound to antibody, assuming tight binding to the IgG. In this case, detergent was added to the sample to reduce the antibody affinity. As a result, in the NMR experiment the researchers were observing the allergen ^1^H-^15^N chemical shift perturbations between bound and free, with the smaller molecule in the free state being the one that was detected. The ratio of bound to free was tuned with the concentration of detergent so that there was a differential reduction or broadening in the NMR signals of those residues in proximity to the antibody compared to the free protein. The broadening is due to increased relaxation of the NMR signal due to the large fully ^1^H labeled antibody binding to the antigen (138). This same effect is noted below in other experiments. In the Der f 2 study, the differential exchange broadening data provided results that mapped the epitopes to two disparate regions of the protein, consistent with the simultaneous binding of the two antibodies. A potential disadvantage of this technique is that it requires empirical tuning of the solvent conditions, which may or may not be applicable to all systems.

**Figure 9:**
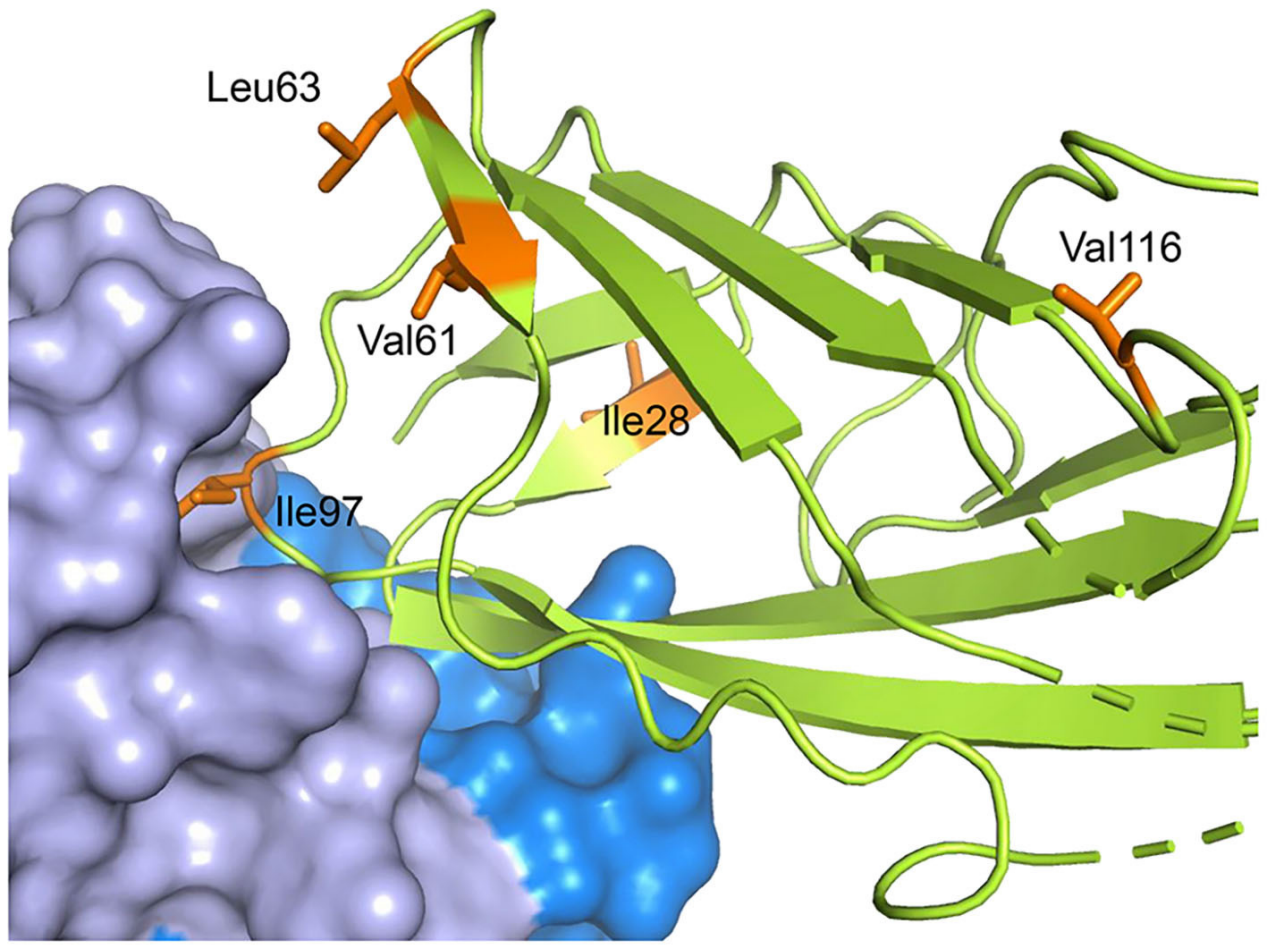
Residues both near and far from the epitope can be affected by antibody binding. The crystal structure of Der p 2 in complex with the murine IgG mAb 7A1 is shown with Der p 2 rendered in green with specific methyl residues highlighted in orange. The mAb 7A1 heavy and light chains are rendered as blue and lavender surfaces, respectively. The shift of methyl resonances of the orange residues upon complex formation were measured by NMR. The figure shows that residues proximal and distal from the epitope can be affected by binding to an antibody.

Instead of looking at fragments of the antibody-allergen complex, or the free allergen in exchange with complex, it is also possible to utilize whole antibodies, but this requires another compromise in the labeling scheme. At the very high molecular weights of allergen-antibody complexes, usually only methyl groups are still observable in a background of otherwise ^2^H labeled proteins (132). Focusing on only labeling methyl groups in the allergen restricts the number of probes available for epitope mapping to the methyl groups of Val, Leu and Ile. The effectiveness of this was demonstrated for [U-^2^H, ^1^H,^13^C-methyl Val, Leu, Ile] Der p 2 bound to an scFv fragment of mAb 7A1 (58). The data showed relaxation broadening for Ile-97 in the allergen, which was directly in contact with the scFv, and chemical shift perturbations for V63 and L61, which were adjacent to the epitope as described in a crystal structure (Figure 9). This is again consistent with previous observations that close proximity to the ^1^H antibody causes broadening or a disappearance of signal, and distal residues can also experience chemical shift perturbations (138).

Methyl labeled Der p 2 as described above was successfully applied to study 4 human IgE mAb epitopes and 3 murine IgG mAb epitopes (127). Similar data observations of broadening and chemical shift perturbations were analyzed to map the epitope regions. Since the epitope data is sparse, being only derived from a few methyl probes, it needs to be interpreted with caution. For each antibody, the data indicated that few residues in close proximity were broadened or perturbed, and this was consistent with previous mutagenesis studies or data from Der p 2 isoforms. The NMR data were in agreement with the relative epitope mapping obtained using competitive and direct antibody binding immunoassays, which demonstrated which epitopes did or did not overlap.

While these data are an impressive first mapping of human IgE epitopes, three important drawbacks of the technique need to be mentioned. First, it requires monoclonal antibodies, of which human IgE are very difficult to clone from patients. Although this technique may be applicable to polyclonal antibodies, its effectiveness remains to be demonstrated in this case. ^1^H-^15^N labeling of Art v 1 and Bet v 1 was combined with either pooled allergic sera, or individual allergic sera, respectively, but the NMR results were nebulous (139–141). Second, the methyl labeling is expensive, typically $1,000 per liter of bacterial expression culture. Thus, high expression levels of the protein are needed to be cost effective. And third, as mentioned above, the distribution of sparse methyl groups may not be ideal for all allergens to get good epitope data. The paucity of data also requires a rather generous interpretation of which residues might be directly involved in the epitope. Hence, the epitopes proposed from these NMR data likely include more residues than the ones that are directly observed contacting the antibody in a crystal structure and should be further tested for functional importance.

In summary, a variety of NMR techniques and labeling schemes have been applied for allergen epitope mapping. In each case, atoms or residues specific to the epitope were successfully identified.

## Future Directions

Experimental epitope mapping of IgE antibodies on allergens originated ~30 years ago with the identification of mostly linear epitopes. Several breakthroughs have allowed the identification of conformational epitopes. These epitopes are the most common on allergens, especially on allergens for which exposure occurs by inhalation. Techniques such as recombinant technology, X-ray crystallography and nuclear magnetic resonance were developed and used for the determination of structures of allergen-antibody complexes. These advances required preparation of pure and homogeneous allergens and monoclonal antibodies. Initially, mostly IgG antibodies that inhibit IgE antibody binding were used as surrogates of IgE and fragmented for epitope mapping. Another approach led to the isolation of IgE antibody constructs using phage display technology. Only more recently, single cell antibody sequencing and human hybridoma technology are opening a new era of epitope mapping that will allow direct visualization of allergen-IgE antibody interactions in detail. Other technologies such as cryo-electron microscopy and labeling with mass spectrometry will also contribute to epitope mapping with less demanding protein amounts. Moreover, the experimental results allow for a significant development of many computational approaches to identify and/or analyze paratopes and epitopes (142). For example, approaches used in image recognition, like Zernike moments, were shown to be very promising in predicting B-cell epitopes (143–145). Therefore, we expect that computational methods will start to play a more important role in studies of interactions between antibodies and allergens. Ultimately, identification of IgE antibody binding epitopes associated with the human IgE repertoire will contribute to understanding the immune response to allergens and will lead to the design of modified recombinant allergens for safer and more effective immunotherapy.

## Author Contributions

AP designed, wrote, and edited the review. She especially wrote the sections about IgE epitope mapping. GM wrote the sections associated with NMR and cryo-EM. MC analyzed crystal structures of allergen-antibody complexes and wrote sections about X-ray crystallography and mass spectrometry. GM and MC also contributed to editing the manuscript. All authors contributed to the article and approved the submitted version.

## Conflict of Interest

AP was employed by the company Indoor Biotechnologies, Inc. She is the contact PI of the R01 Award that funded this manuscript. The remaining authors declare that the research was conducted in the absence of any commercial or financial relationships that could be construed as a potential conflict of interest. The handling editor declared a past co-authorship with one of the authors AP.
